# Supplementary material for: A novel probe to assess cytosolic entry of exogenous proteins
Source: Nat Commun. 2018 Aug 6;9:3104. doi: 10.1038/s41467-018-05556-z (PMC6079096; doi:10.1038/s41467-018-05556-z)
Supplement: Supplementary file 1 — Supplementary Information [file 41467_2018_5556_MOESM1_ESM.pdf]

Supplementary Information

A novel probe to assess cytosolic entry of exogenous proteins

Lu et al.

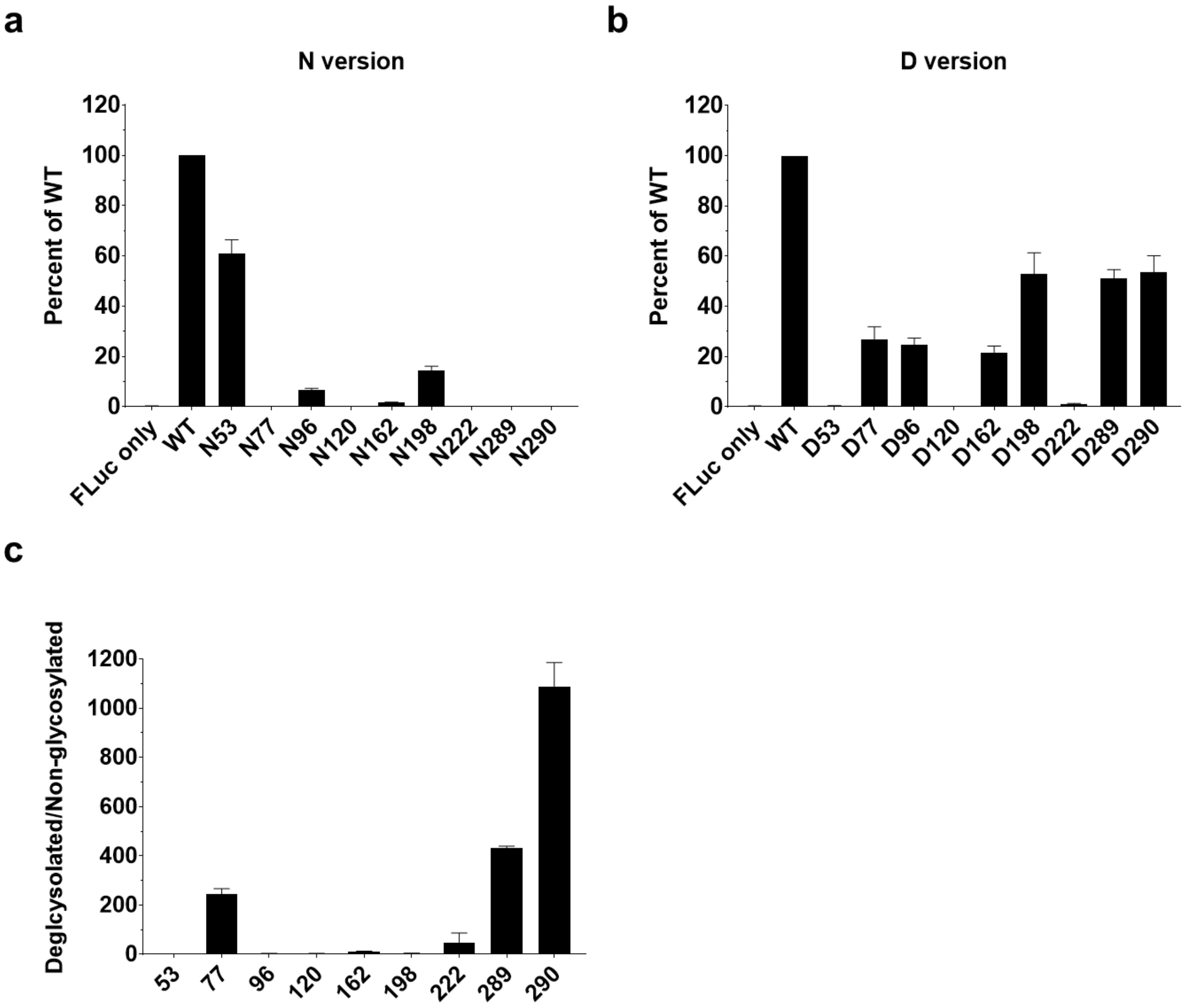

Supplementary Figure 1. Cytosolic selection of deglycosylation-dependent RLuc variants.

RLuc variants were expressed in the cytosol of 293T cells for an initial screen of deglycosylation-dependent RLuc (ddRLuc) activity. 36 h post transfection, cells were lysed and assayed for luciferase activity. (a) Luminescence generation from the 'glycosylated' (N) version of RLuc variants. (b) Luminescence generation from the 'deglycosylated' (D) version of RLuc variants. (c) Fold difference in luminescence generated by the D versions versus the N versions. Bars represent the mean+/-s.d. of one representative experiment with triplicates. Representative data of three independent experiments are shown.

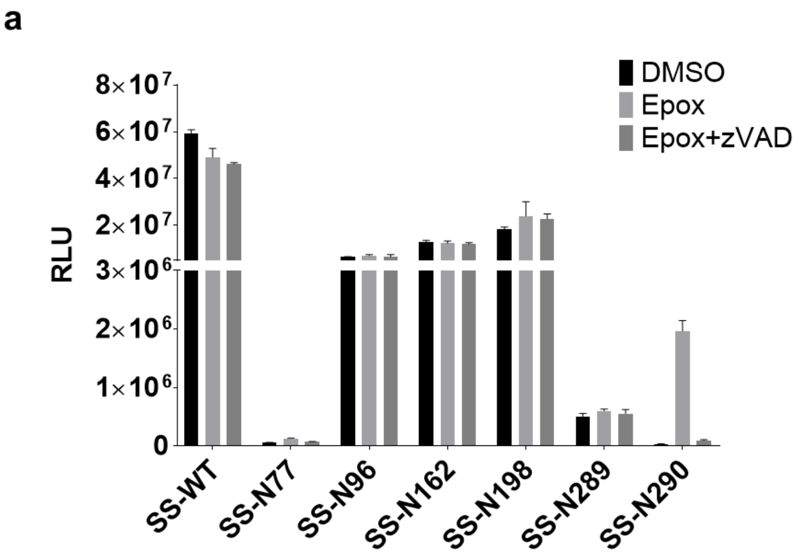

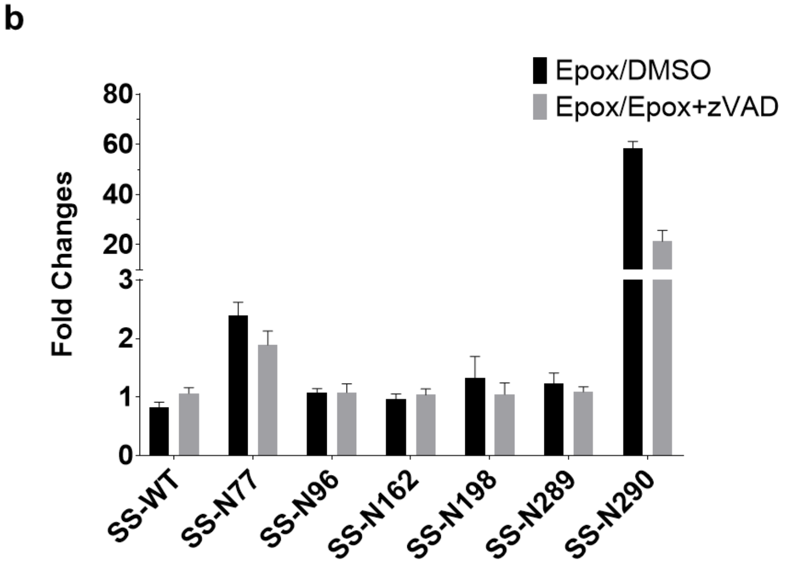

Supplementary Figure 2. Confirmation of deglycosylation-dependent activity after proteasome and NGLY1 inhibition.

RLuc variants (N-version) incorporating a signal sequence (SS-NxS/T) were transfected into 293T cells. 36 h post transfection, cells were incubated with DMSO, 200 nM Epox, or a combination of 200 nM Epox and 20 μM zVAD for 6 h before being lysed and subjected to luciferase assays. (a) RLU generated from different SS-RLuc variants. (b) Fold difference between Epox-treated samples and others. Bars represent the mean+/-s.d. of one representative experiment with triplicates. Representative data of three independent experiments are shown.

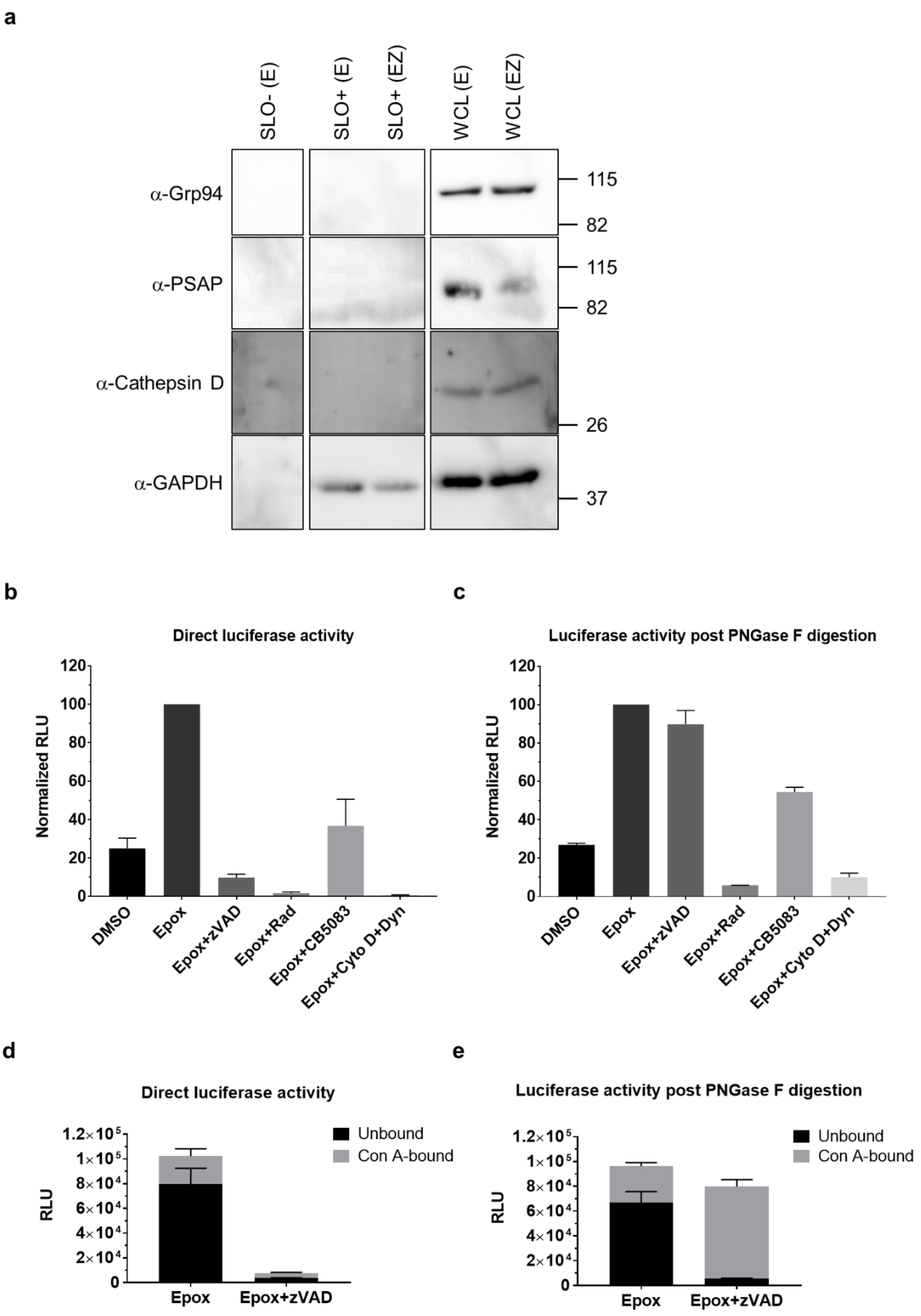
a
SLO- (E)
SLO+ (E)
SLO+ (EZ)
WCL (E)
WCL (EZ)
α-Grp94
α-PSAP
α-Cathepsin D
α-GAPDH
115
82
115
82
26
37
b
Direct luciferase activity
Normalized RLU
120
100
80
60
40
20
0
DMSO
Epox
Epox+zVAD
Epox+Rad
Epox+CB5083
Epox+Cyto D+Dyn
c
Luciferase activity post PNGase F digestion
Normalized RLU
120
100
80
60
40
20
0
DMSO
Epox
Epox+zVAD
Epox+Rad
Epox+CB5083
Epox+Cyto D+Dyn
d
Direct luciferase activity
RLU
1.2×10⁵
1×10⁵
8×10⁴
6×10⁴
4×10⁴
2×10⁴
0
Unbound
Con A-bound
Epox
Epox+zVAD
e
Luciferase activity post PNGase F digestion
RLU
1.2×10⁵
1×10⁵
8×10⁴
6×10⁴
4×10⁴
2×10⁴
0
Unbound
Con A-bound
Epox
Epox+zVAD

Supplementary Figure 3. Post deglycosylation ddRLuc-Fc luminescence is cytosolic.

293T-FcRγIIA-K^b were fed ddRLuc-Fc-bound 3 μm latex beads for phagocytosis and incubated at 37 °C for 8 h, followed by cytosol purification after Streptolysin O (SLO) permeabilization as described in Methods.

(a) The purity of the extracted cytosol was assessed by western blot for the ER protein Grp94, the endosomal, lysosomal/phagosomal proteins prosaposin (PSAP) and cathepsin D, and the cytosolic protein GAPDH (E= Epox, EZ= Epox+zVAD).

(b-c) The isolated cytosol was directly assessed for luciferase activity to quantify the amount of dislocated deglycosylated ddRLuc-Fc (labeled "Direct luciferase activity"). To reveal the total amount of dislocated ddRLuc-Fc, purified cytosol was digested by PNGase F *in vitro* and then assessed for luciferase assay (labeled "Luciferase activity post PNGase F Digestion"). RLU on the *y* axes are normalized to the activity obtained with Epox alone (set to 100). Drugs were used at the following concentrations: 200 nM Epox, 20 μM zVAD, 32 μM Rad, 1 μM CB5083, 2.5 μg mL^{-1} Cyto D plus 100 μM Dyn.

(d-e) Isolated cytosol was incubated with Concanavalin A (Con A)-Sepharose at 4 °C for 2 h to pull down glycosylated protein. The Con A beads were then washed and resuspended in PBS. The direct luciferase activity (d) and total luciferase activity post PNGase F digestion (e) in the unbound fraction (delycosylated) and the Con A-bound (glycosylated) activity are shown.

In b-e, bars represent the mean+/-s.d. of one representative experiment with duplicates. Representative data of three independent experiments are shown.

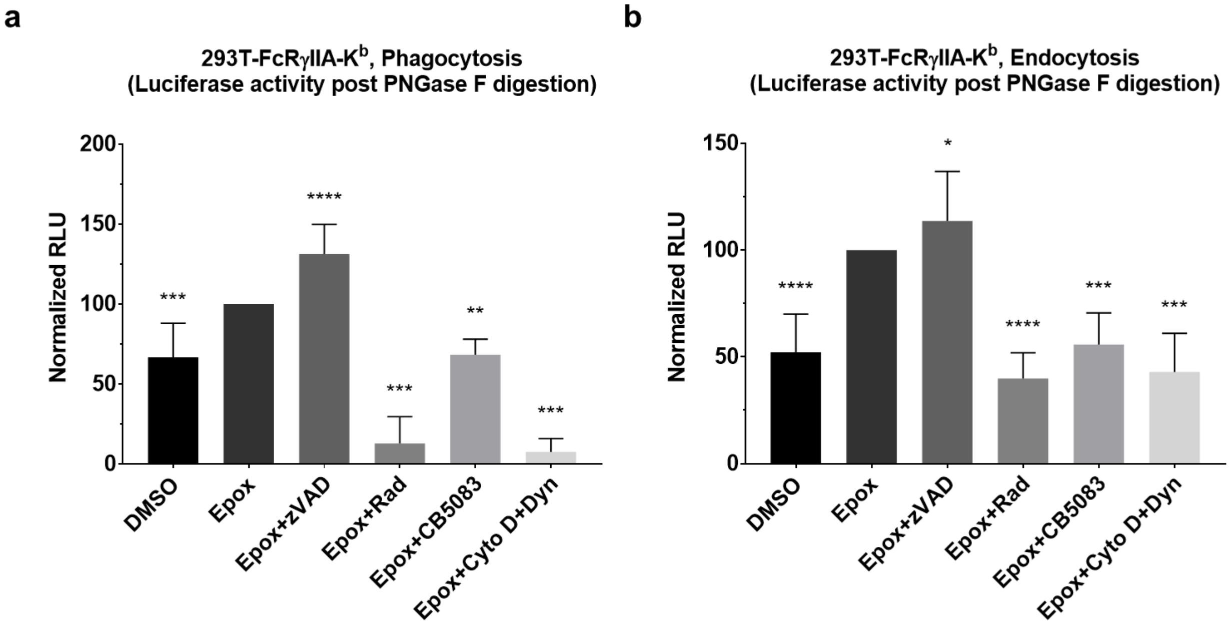

Supplementary Figure 4. Detection of total intracellular ddRLuc-Fc in 293T-FcRγIIA-K^b cells.

293T-FcRγIIA-K^b cells were fed ddRLuc-Fc-bound 3 μm latex beads for phagocytosis (a) or soluble ddRLuc-Fc at 100 μg mL^{-1} for endocytosis (b) in the presence of various inhibitors and incubated at 37 °C for 6 h. Cells were washed and lysed as described in Methods. Cell lysates were first digested by PNGase F and then immediately assayed for luciferase activity to quantify total intracellular ddRLuc-Fc (labeled "Luciferase activity post PNGase F digestion"). The values for RLU are normalized to Epox-treated samples (set to 100). Drugs were used at the following concentrations: 200 nM Epox, 20 μM zVAD, 32 μM Rad, 1 μM CB5083, 2.5 μg mL^{-1} Cyto D plus 100 μM Dyn. Bars represent the mean+/-s.d. of at least four independent experiments per treatment (paired two-tailed *t*-test, *p<0.05; **p<0.01; ***p<0.001; ****p<0.0001). Data were obtained in the same experiments shown in Fig. 2a-b.

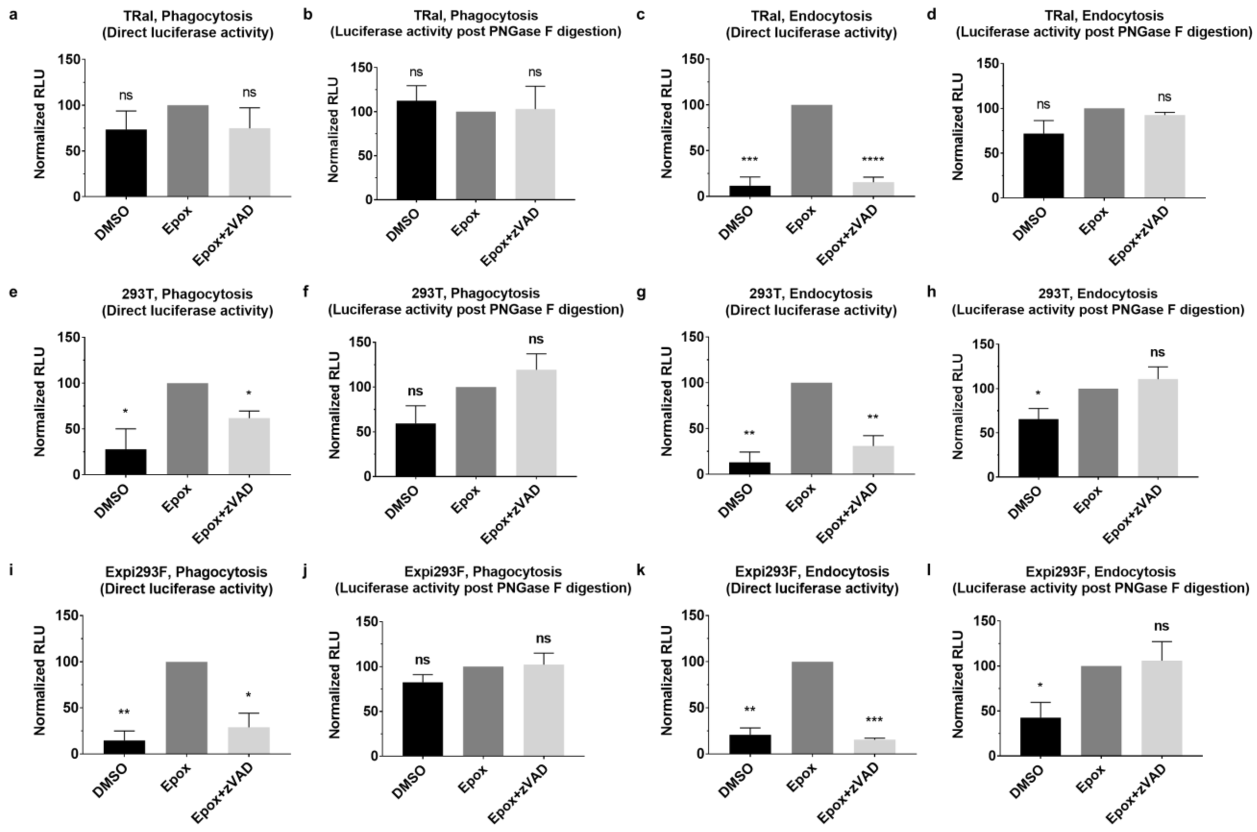

Supplementary Figure 5. ddRLuc-Fc dislocation in a variety of cell lines.

TRal (a-d), 293T (e-h), and Expi293F (i-l) cell lines were fed ddRLuc-Fc-bound 3 μm latex beads for phagocytosis (a-b, e-f, i-j) or 100 μg mL^{-1} soluble ddRLuc-Fc for endocytosis (c-d, g-h, k-l) in the presence of various drugs (DMSO only, Epox, or a combination of Epox with 20 μM zVAD). Epox were used at the following concentrations: 800 nM Epox for TRal cells, 200 nM Epox for 293T and Expi293F cells. Cells were washed and lysed 6 h post feeding. Cell lysate was directly assessed for luciferase assay to quantify dislocated ddRLuc-Fc (a, c, e, g, i, and k, labeled "Direct luciferase activity") as described in Fig. 2 or first digested by PNGase F and then assessed for luciferase assay to quantify total intracellular ddRLuc-Fc (b, d, f, h, j, and l, labeled "Luciferase activity post PNGase F digestion") as described Supplementary Fig. 4. Bars represent the mean+/-s.d. of three independent experiments per treatment (paired two-tailed *t*-test, *p<0.05; **p<0.01; ***p<0.001; ****p<0.0001; ns, not significant).

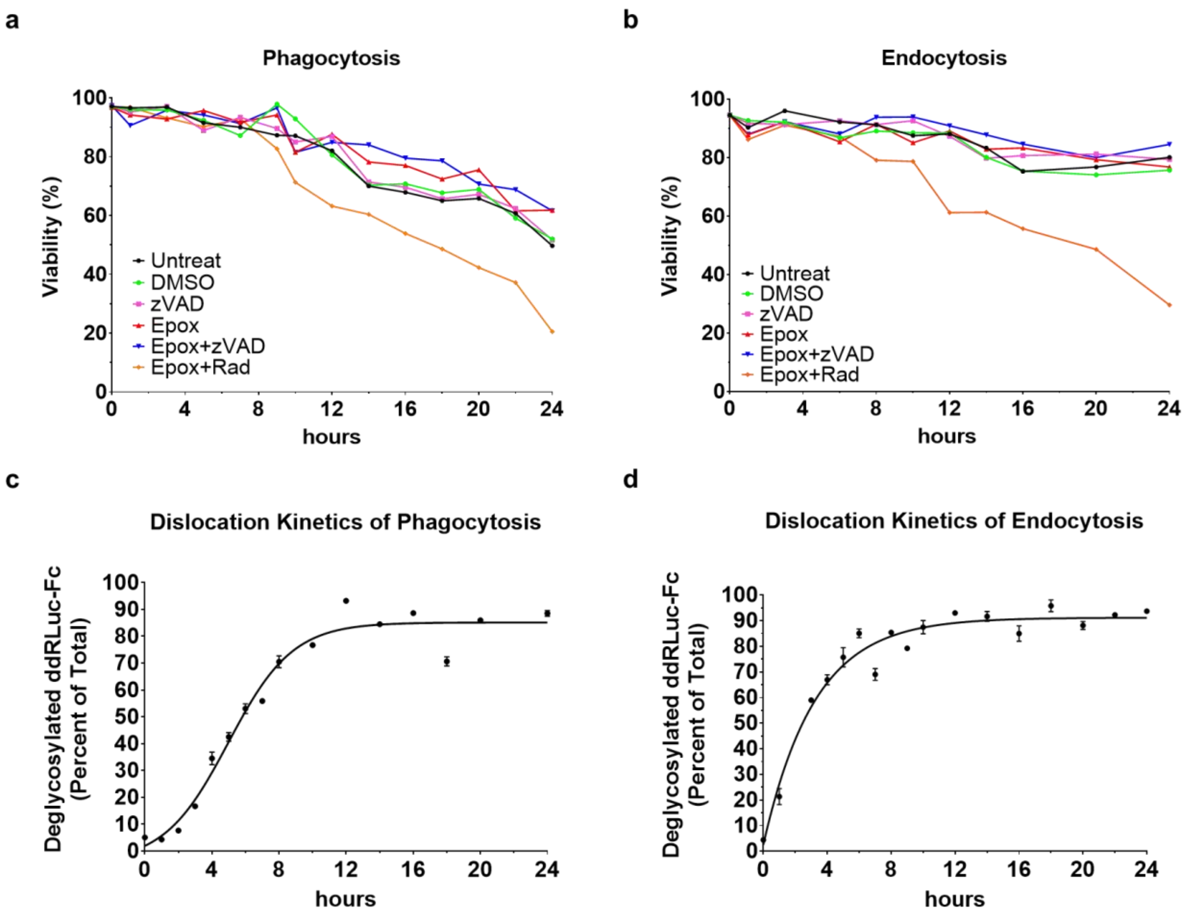

Supplementary Figure 6. Cell viability and the rate of deglycosylation of ddRLuc-Fc after internalization in the presence of various drugs.

(a, b) Viability of 293T-FcRγIIA-K^b cells was determined by Trypan blue staining after phagocytosis (a) and endocytosis (b) in the presence of various drugs (200 nM Epox, 20 μM zVAD, or 32 μM Rad).

(c, d) Kinetics of ddRLuc-Fc deglycosylation after phagocytosis (c) and endocytosis (d) by 293T-FcRγIIA-K^b cells in the presence of 200 nM Epox. 8 μL of cell lysate was incubated at 37 ˚C for 10 min with or without PNGase F, followed by luciferase assays. The RLU from undigested samples was calculated as a percentage of the RLU from PNGase F digested samples. This serves as an indicator of the percentage of dislocated and deglycosylated ddRLuc-Fc out of total intracellular ddRLuc-Fc over time. Points represent the mean+/-s.d. of one representative experiment with duplicates.

Representative data of three independent experiments are shown and the data were from the same experiment shown in Fig. 3.

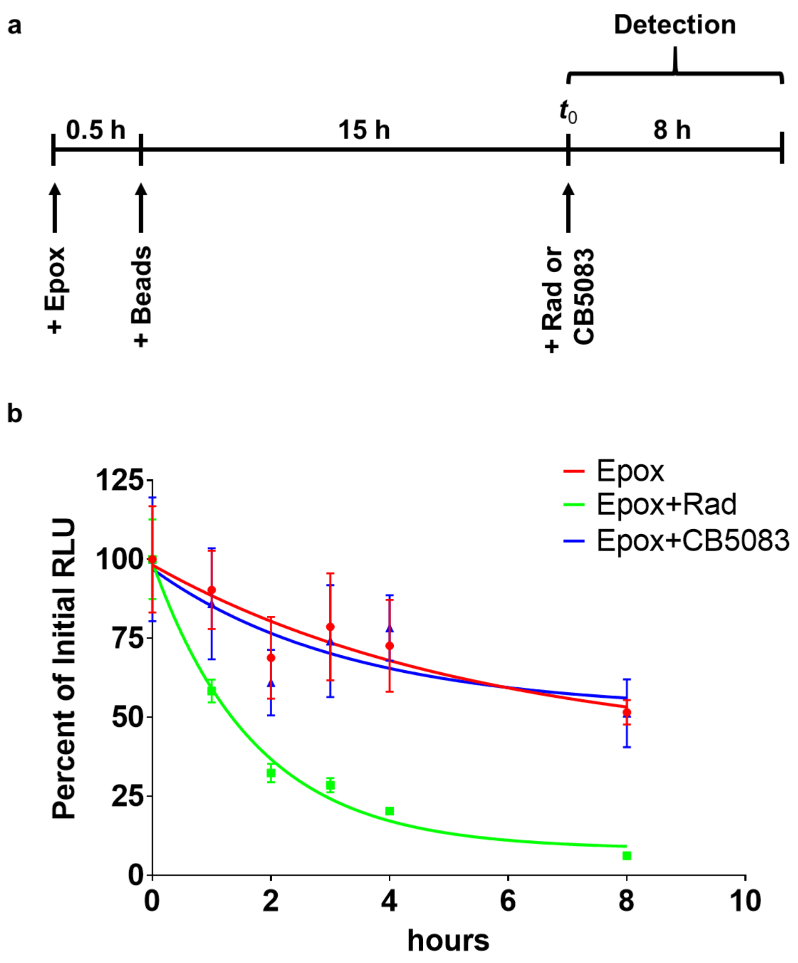

Supplementary Figure 7. Hsp90 stabilizes the dislocated ddRLuc-Fc in the cytosol.

(a) Schematic description of the experiment. 293T-FcRγIIA-K^b cells were treated with Epox (200 nM) for 0.5 h before being fed ddRLuc-Fc-bound 3 μm latex beads for phagocytosis. Cells were incubated at 37 °C in the presence of Epox for 15 h, followed by the addition of radicicol (Rad, 16 μM) or CB5083 (1 μM) to the cell culture (t_0). Cell lysates were then prepared, processed and assayed for luciferase assay at various time after addition of Rad or CB5083. (b) Kinetics of decay in luciferase activity. RLU values were normalized to the value at t_0 (set to 100%). Points represent the mean+/-s.d. of one representative experiment with four replicates. Representative data of two independent experiments are shown.

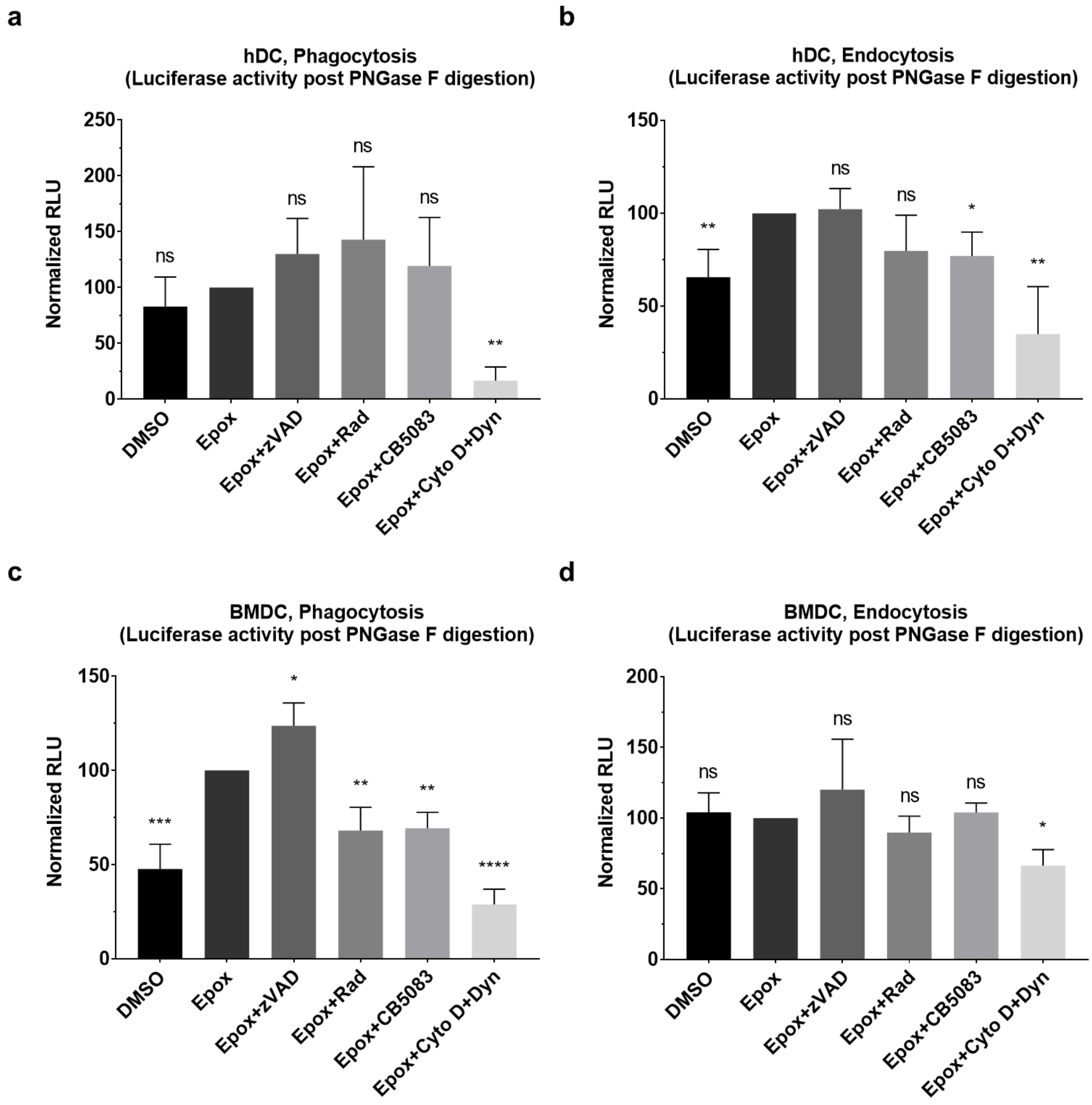

Supplementary Figure 8. Total ddRLuc-Fc internalized by primary dendritic cells.

Primary dendritic cells were incubated with ddRLuc-Fc-bound 3 μm latex beads for phagocytosis (a, c) or soluble ddRLuc-Fc at 100 μg mL^{-1} for endocytosis (b, d). Cell lysates were prepared, digested with PNGase F and analyzed as described in Supplementary Fig. 4. Human DCs (hDCs, a-b) were harvested 6 h after phagocytosis/endocytosis while mouse bone marrow-derived DCs (BMDCs, c-d) were harvested after 3 h. Drugs were used at the following concentrations: 800 nM Epox, 20 μM zVAD, 32 μM Rad, 1 μM CB5083, 2.5 μg mL^{-1} Cyto D plus 100 μM Dyn. Bars represent the mean+/-s.d. of at least three independent experiments per treatment (paired two-tailed *t*-test, *p<0.05; **p<0.01; ***p<0.001; ****p<0.0001; ns, not significant). Data were from the same experiments shown in Fig. 4a-d.

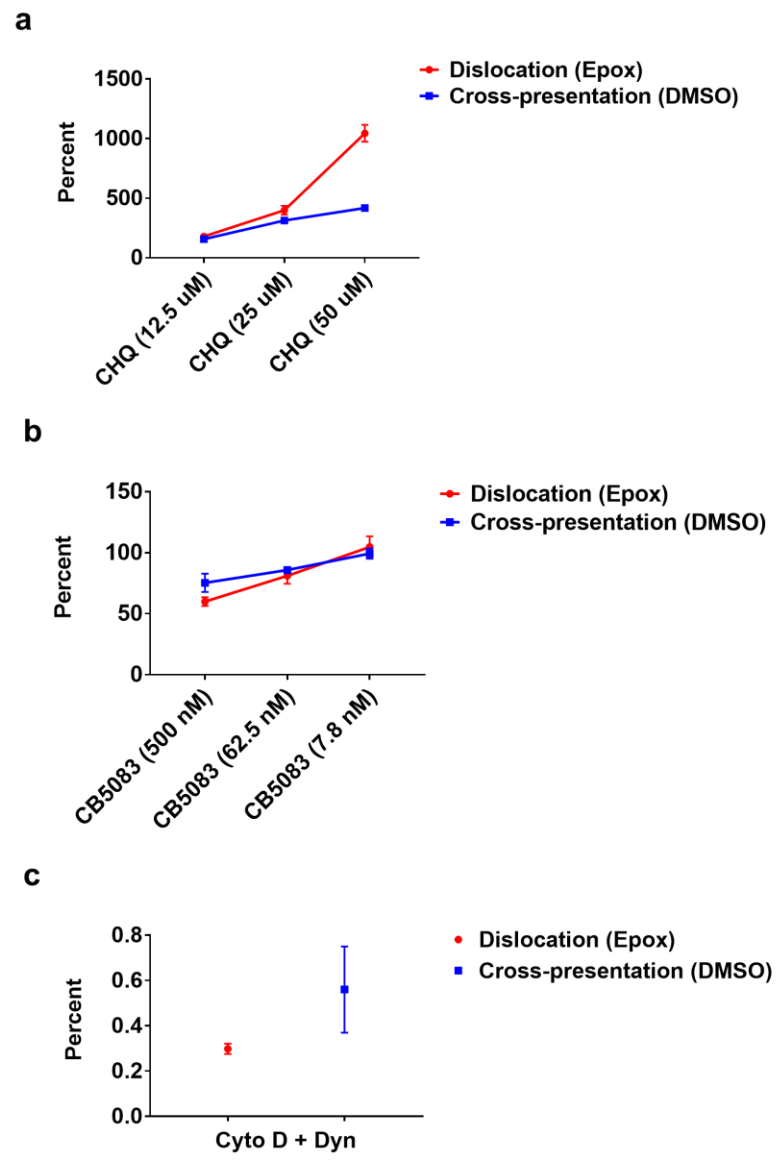

Supplementary Figure 9. Effects of chloroquine, CB5083 and Cytochalasin D/Dynasore on dislocation and cross-presentation.

293T-FcRγIIA-K^b cells were fed ddRLuc-Fc^{OVA}-bound 3 μm latex beads for phagocytosis and incubated at 37 °C for 8 h in the presence or absence of the various inhibitors. One set of cells were incubated in the presence of Epox with or without other inhibitors and assayed for luciferase activity as described in Fig. 2, and the other set of cells were incubated with DMSO with or without other inhibitors and assessed for cross-presentation as described in Methods. The values presented on the *y* axes are normalized to Epox alone for dislocation (luciferase activity, red lines) and no drug (DMSO only) for cross-presentation, measured by IL-2 secretion by B3Z cells activated by the cells treated with various inhibitors (blue lines). (a) Chloroquine (CHQ) titration; (b) CB5083 titration; (c) 2.5 μg mL^{-1} Cyto D and 100 μM Dyn. Points represent the mean+/-s.d. of one representative experiment with triplicates. Representative data from three independent experiments are shown.

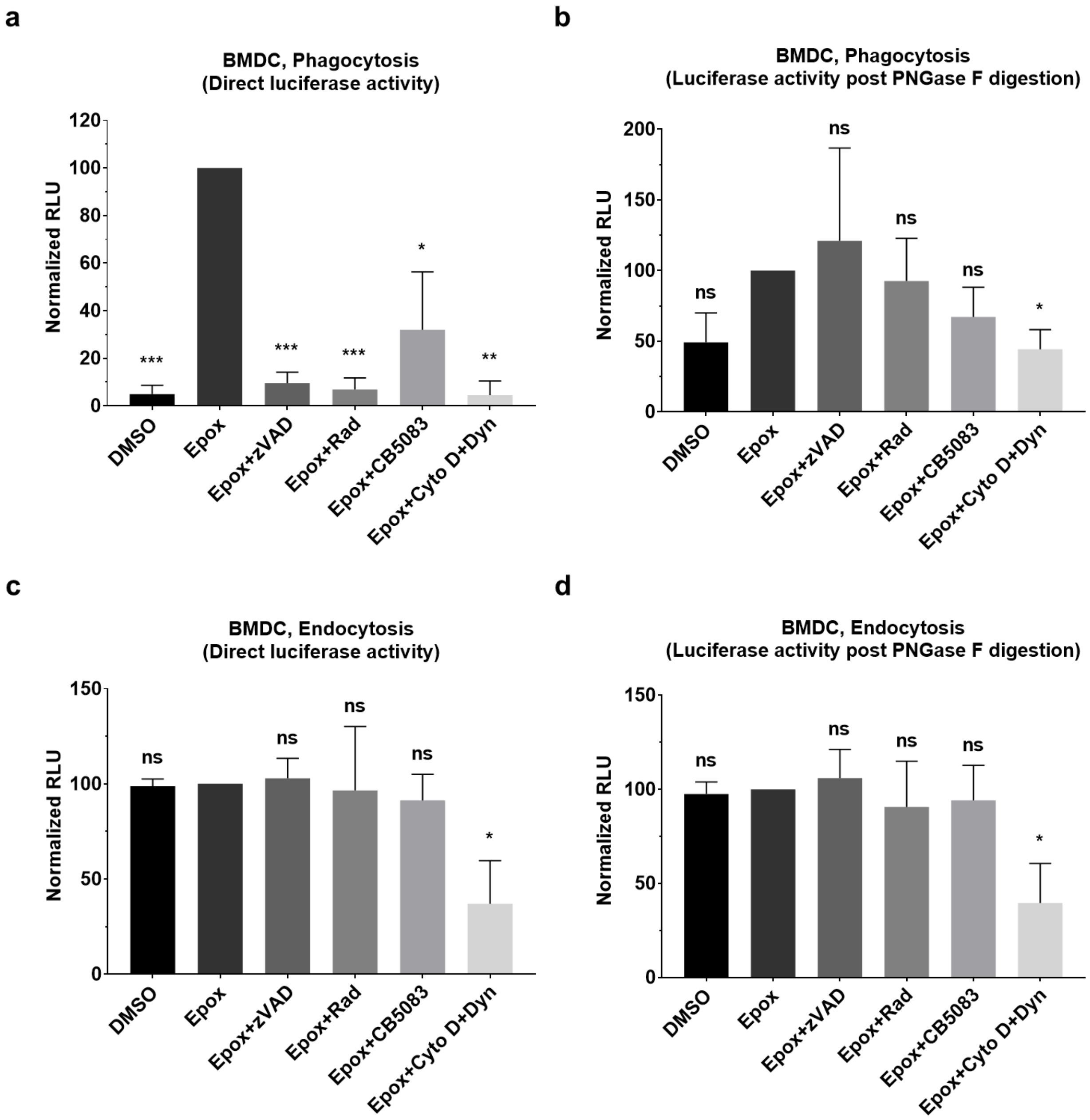

Supplementary Figure 10. ddRLuc-Fc^{CHO-} dislocation in BMDCs.

BMDCs were fed ddRLuc-Fc^{CHO-}-bound 3 μm latex beads for phagocytosis (a-b) or soluble ddRLuc-Fc^{CHO-} at 100 μg mL^{-1} for endocytosis (c-d). BMDCs were washed and lysed 3 h post feeding. Cell lysate was processed and analyzed as described in Fig. 2 for measuring direct luciferase activity (a, c) and as described in Supplementary Fig. 4 for quantifying total luciferase activity post PNGase F digestion (b, d). Drugs were used at the following concentrations: 800 nM Epox, 20 μM zVAD, 32 μM Rad, 1 μM CB5083, 2.5 μg mL^{-1} Cyto D plus 100 μM Dyn. Bars represent the mean+/-s.d. of three independent experiments per treatment (paired two-tailed *t*-test, *p<0.05; **p<0.01; ***p<0.001; ns, not significant).

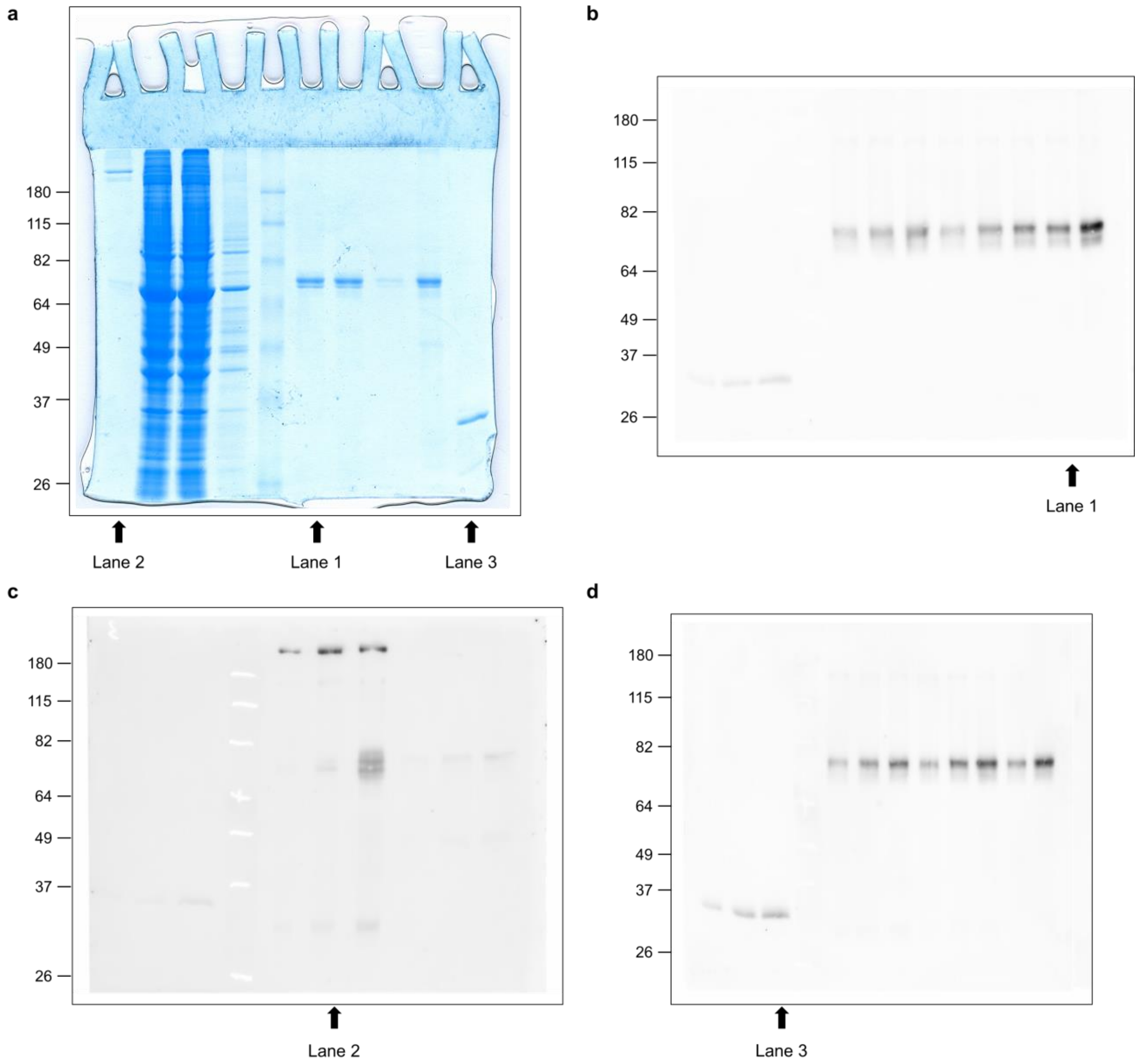

Supplementary Figure 11. Uncropped gel of Figure 1d.

(a) Uncropped gel of Coomassie blue gel. (b-d) Uncropped gel of Western blot. Arrows indicate the lanes shown in Figure 1d.

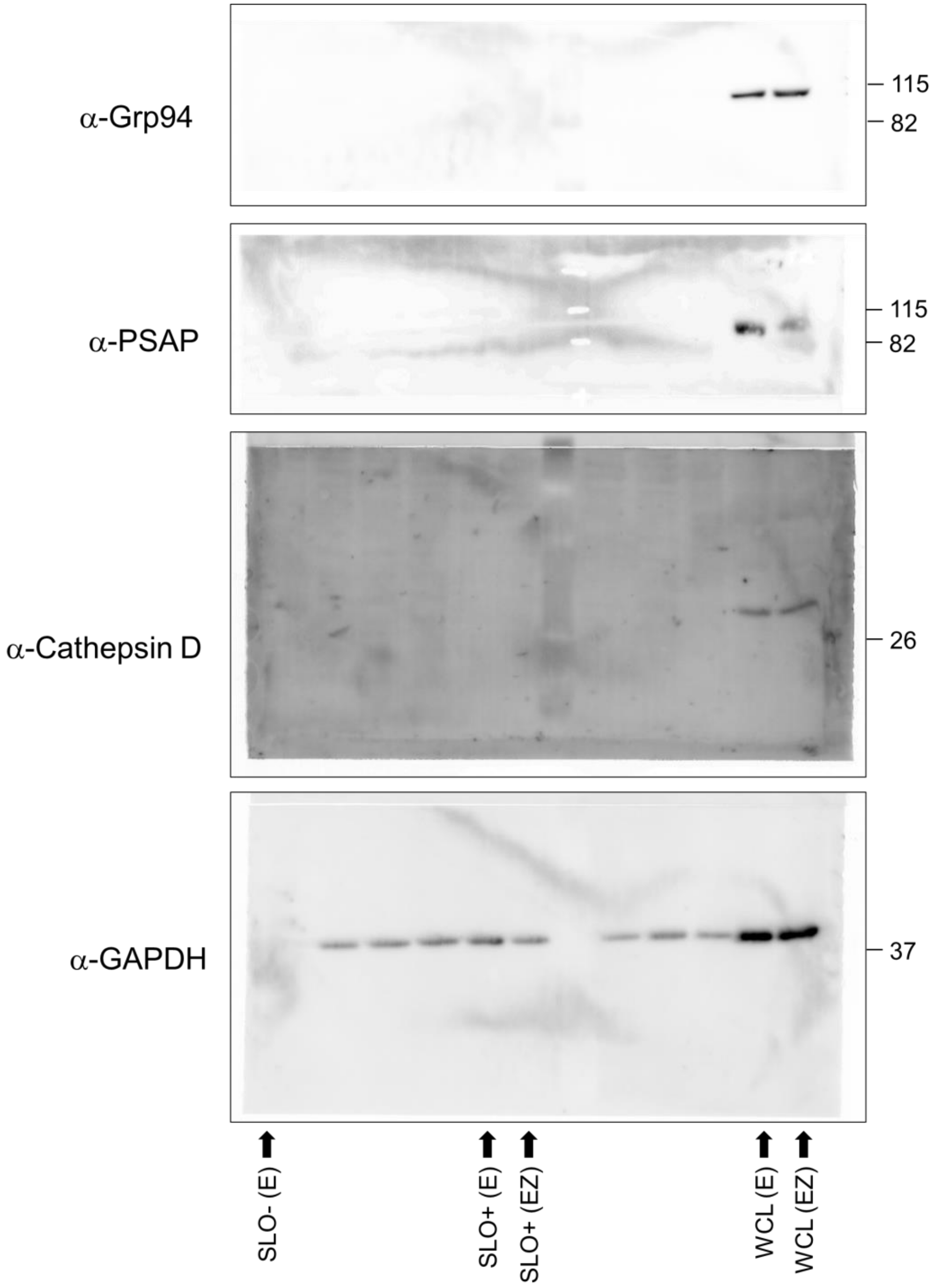

Supplementary Figure 12. Uncropped gel of Supplementary Figure 3a.

Membranes were cut into individual sections probed with different antibodies. Arrows indicate the lanes shown in Supplementary Figure 3a. Other lanes contain purified cytosol from cells treated with different drugs.

Supplementary Table 1. Representative PNGase F digested RLU of DMSO and Epox treated samples from different cell types.

RLU [1]	Phagocytosis		Endocytosis	
	DMSO (x 10^4)	Epox (x 10^4)	DMSO (x 10^4)	Epox (x 10^4)
**293T-FcRγIIA-K^b [2]**	9.53	30.53	0.50	1.37
**TRaI [3]**	13.50	14.55	0.74	1.10
**293T [4]**	2.98	3.67	0.22	0.35
**Expi293F [5]**	1.42	1.65	0.46	0.83
**hDC [6]**	3.24	3.97	3.76	9.27
**BMDC [7]**	2.68	5.85	246.06	239.68
**BMDC [8] †**	2.07	5.38	266.24	260.34

1. RLU was measured from 8 μL total cell lysate after 10 min digestion at 37 °C by PNGase F (consider protein destabilization/loss during this incubation). Mean value of background-subtracted normalized RLU from a representative experiment is shown.

2. 2 x 10^5 293T-FcRγIIA-K^b cells were assayed at 6 h after feeding, and Epox was used at 200 nM for proteasome inhibition.

3. 2 x 10^5 TRaI cells were assayed at 6 h after feeding, and Epox was used at 800 nM.

4. 2 x 10^5 293T cells were assayed at 6 h after feeding, and Epox was used at 200 nM.

5. 2 x 10^5 Expi293F cells were assayed at 6 h after feeding, and Epox was used at 200 nM.

6. 2 x 10^5 hDCs were assayed at 6 h after feeding, and Epox was used at 800 nM.

7. 5 x 10^5 BMDCs were assayed at 3 h after feeding, and Epox was used at 800 nM.

8. † 5 x 10^5 BMDCs were assayed at 3 h after being fed with ddRLuc-Fc^{CHO-}, and Epox was used at 800 nM.

Supplementary Table 2. RLU generated from glycosylated ddRLuc-Fc and deglycosylated ddRLuc-Fc.

ng ddRLuc-Fc	RLU PNGase F- (glycosylated) (x 10^4)	RLU PNGase F+ (deglycosylated) (x 10^4)
0	0.08	0.11
1.25	0.11	2.76
2.5	0.21	8.18
5	0.47	19.88
10	1.07	49.93
25	2.59	109.28
50	6.32	289.75
100	17.75	921.98
200	53.22	3584.83
300	87.39	6279.67

This table shows the mean value of RLU generated from 10 different batches of purified ddRLuc-Fc after 10 min incubation at 37 ˚C with or without PNGase F.
